# Structure-guided screening strategy combining surface plasmon resonance with nuclear magnetic resonance for identification of small-molecule Argonaute 2 inhibitors

**DOI:** 10.1371/journal.pone.0236710

**Published:** 2020-07-31

**Authors:** Toshimasa Harumoto, Atsuko Sato, Yuki Takayama, Hikaru Miyagi, Jun-ichi Saito, Fumikazu Shinohara

**Affiliations:** 1 Research Functions Unit, R&D Division, Kyowa Kirin Co., Ltd., Machida-shi, Tokyo, Japan; 2 Research Functions Unit, R&D Division, Kyowa Kirin Co., Ltd., Nagaizumi-cho, Suntou-gun, Shizuoka, Japan; 3 Research Functions Unit, R&D Division, Kyowa Kirin Co., Ltd., Otemachi, Chiyoda-ku, Tokyo, Japan; The University of Tokyo, JAPAN

## Abstract

Argonaute (AGO) proteins are the key component of the RNA interference machinery that suppresses gene expression by forming an RNA-induced silencing complex (RISC) with microRNAs (miRNAs). Each miRNA is involved in various cellular processes, such as development, differentiation, tumorigenesis, and viral infection. Thus, molecules that regulate miRNA function are expected to have therapeutic potential. In addition, the biogenesis of miRNA is a multistep process involving various proteins, although the complete pathway remains to be elucidated. Therefore, identification of molecules that can specifically modulate each step will help understand the mechanism of gene suppression. To date, several AGO2 inhibitors have been identified. However, these molecules were identified through a single screening method, and no studies have specifically evaluated a combinatorial strategy. Here, we demonstrated a combinatorial screening (SCR) approach comprising an *in silico* molecular docking study, surface plasmon resonance (SPR) analysis, and nuclear magnetic resonance (NMR) analysis, focusing on the strong binding between the 5'-terminal phosphate of RNA and the AGO2 middle (MID) domain. By combining SPR and NMR, we identified binding modes of amino acid residues binding to AGO2. First, using a large chemical library (over 6,000,000 compounds), 171 compounds with acidic functional groups were screened using *in silico* SCR. Next, we constructed an SPR inhibition system that could analyze only the 5'-terminal binding site of RNA, and nine molecules that strongly bound to the AGO2 MID domain were selected. Finally, using NMR, three molecules that bound to the desired site were identified. The RISC inhibitory ability of the “hit” compounds was analyzed in human cell lysate, and all three hit compounds strongly inhibited the binding between double-stranded RNA and AGO2.

## Introduction

MicroRNAs (miRNAs) are small non-coding RNAs that regulate gene expression and are known to play a role in various cellular functions, such as development and differentiation [1–3]; however, miRNAs do not function by themselves but bind to certain proteins to carry out their functions. Typically, primary miRNA (pri-miRNA) is transcribed by polymerase II, which has one or more stem-loop structures. In the nucleus, the pri-miRNA is further cleaved by Drosha and DiGeorge syndrome critical region 8 (DGCR8) to produce pre-miRNAs. Following transportation to the cytoplasm by Exportin-5, pre-miRNA is cleaved by the RNase III enzyme Dicer, generating double-stranded RNA (miRNA/miRNA* duplex). This double-stranded RNA is incorporated into Argonaute (AGO), followed by removal of the passenger strand to form the RNA-induced silencing complex (RISC), which then suppresses gene expression [4,5].

AGO is the central protein in the RNA interference (RNAi) machinery and is highly conserved from yeast to mammals [6]. In mammals, four AGO proteins (AGO1-4) have been identified, of which only AGO2 has slicing activity [7]. Structurally, AGO proteins are composed of four domains, namely, the amino-terminal domain (N-domain), the middle (MID) domain, the Piwi-Argonaute-Zwille (PAZ) domain, and the P-element-induced wimpy testis (PIWI) domain. Seven nucleotides from the 5' end of the antisense strand bind to the MID domain, and two nucleotides from the 3' end bind to the PAZ domain. Each miRNA recognizes 7-8 nucleotides of the target mRNA, such that one miRNA can recognize a large number of mRNAs (~100) and regulate various functions *in vivo*.

Some miRNAs are known to be involved in diseases. For example, miR-21 has been reported as an antiapoptotic factor in cancer cells, and upregulation of miR-21 has been observed in various cancers, such as glioblastomas. Furthermore, inhibition of miR-21 has been shown to suppress progression of malignancy [8]. Further, miR-17-92 is overexpressed in B-cell lymphoma and lung cancer, leading to cancer growth [9,10]. In addition, AGO2 protein levels are associated with cell differentiation. Reduction in AGO2 levels improved retinoic acid-induced myeloid differentiation of acute promyelocytic leukemia cells [11]. The Venezuelan equine encephalitis virus proliferates using the miRNA/AGO machinery, suggesting that inhibition of AGO2 could effectively suppress viral growth [12]. Thus, therapies based on modulation of AGO2 and miRNA are expected to show potential.

In general, to obtain nucleic-acid-protein inhibitors is considered challenging. Nevertheless, inhibitors of RAD52-ssDNA interaction have been identified by FRET-based high-throughput screening [13]. Another study identified several inhibitors of the mammalian high mobility group AT-hook 2 (HMGA2) by protein–DNA interaction enzyme-linked immunosorbent assays (PDI-ELISA) [14]. Therefore, if an optimal screening system can be constructed, nucleic-acid-protein inhibitors can be obtained.

To date, several small molecules that inhibit loading of small RNA into AGO have been identified. Poly-L-lysine inhibits the processing function of Dicer, while 3,6-diamino-10-methylacridinium chloride inhibits the binding between AGO2 and TAR RNA binding protein (TRBP) / RNA helicase A (RHA), which is known to aid the loading of RNA into AGO2 [15]. In a previous study, a fluorescence polarization screening assay was used to identify AGO2 inhibitors, such as oxidopamine-HCL, aurintricarboxylic acid, and suramin sodium salt, from a commercially available compound library [16]. BCI-137, which is also an AGO2 inhibitor, was identified by using a high-throughput molecular docking screening method [17]. However, these AGO2-binding molecules were identified by a single screening method using a medium-scale library (<200,000 compounds), and although the binding of each molecule to AGO2 was confirmed, the binding mode was not fully elucidated.

Here, we combined an *in silico* docking study, surface plasmon resonance (SPR) analysis, and nuclear magnetic resonance (NMR) analysis to identify new small molecules that strongly bind to AGO2 using a large chemical compound library. First, *in silico* screening (SCR) was used to identify strong binding between the 5'-terminal phosphate of RNA and AGO2 [18,19], and small molecules with an acidic functional group were selected. Next, SPR analysis was adopted to identify binding between the 5’ terminus of RNA and the MID domain. SPR is an effective method to analyze direct binding with high throughput. In addition, because it is a cell-free system, evaluation of affinity focusing on the point of interest is possible by using a part of the protein. In this study, we constructed a new SPR system that could analyze only the binding between the 5’ terminus of RNA and the MID domain. Using single-dose and dose-response analyses, nine candidates were successfully screened. Finally, using NMR analysis, we identified three of these nine compounds that bound to the desired pocket. All the “hit” compounds successfully inhibited the RISC loading of small RNA in a HeLa lysate more strongly than BCI-137. Herein, we also propose a new methodology to identify fragments for a nucleic acid-protein binding pocket based on the crystal structure.

## Materials and methods

### Chemical library

Structural data for the compounds available in the chemical library were provided by Namiki Shoji (Tokyo, Japan). Compounds selected by *in silico* SCR were purchased from Namiki Shoji. For the control compounds, adenosine monophosphate (AMP), uridine monophosphate (UMP), cytidine monophosphate (CMP), guanine monophosphate (GMP), and adenosine were purchased from Sigma-Aldrich (St. Louis, MO, USA). BCI-137 was purchased from Enamine (Kyiv, Ukraine).

### Preparation of the AGO2 MID domain

The AGO2 MID domain–expressing vector was constructed by inserting Gln432-Leu578 into the pE-SUMOpro-3 vector (LifeSensors, Malvern, PA, USA). The vector was transformed into *Escherichia coli* BL21(DE3), and a His-tagged AGO2 MID domain was purified using Ni Sepharose 6 Fast Flow (GE Healthcare, Chicago, IL, USA), followed by buffer exchange using a PD-10 Column (GE Healthcare). Following removal of the SUMO tag by SUMO protease 2 (LifeSensors), the AGO2 MID domain was prepared by recovering the flow through the Ni Sepharose 6 Fast Flow solution.

For the NMR analysis, an ^15^N-labeled AGO2 MID domain was prepared by using stable isotope labeled medium Spectra 9 (Cambridge Isotope Laboratories, Tewksbury, MA, USA).

### Preparation of the AGO2 for virtual screening

The crystal structure of the AGO2 (PDB ID 4OLA) was used as a docking template [19]. Hydrogen atoms were added and minimized under the following conditions: Amber 10, extended Hückel method (EHT) force field; temperature, 298 K; pH 7.4; and salt concentration, 0.15 mol/L using protonate 3D in Molecular Operating Environment (MOE) [20]. Two bases from the 5' end of the template structure were deleted, generating an “alpha sphere” using SiteFinder and creating the “dummy atoms” as the docking site. Lastly, the nucleic acid bound to AGO2 was deleted to eliminate the influence of the phosphate charge in the docking process.

### *In silico* SCR

To remove compounds with partial structures and properties that are undesirable as pharmaceuticals from the chemical library, we used Pipeline Pilot [21], a software that can easily manipulate large amounts of data. Filtering compounds was performed using the component of “substructure filter” and “property calculator” in Pipeline Pilot under the following conditions: molecular weight, 200–800; ≤13 heavy atoms; ≤12 rotatable bonds; ≤5 aromatic rings; <3 cyano groups; <2 nitro groups; and no isotopes.

Since compounds having acidic functional groups are expected to bind strongly with the AGO2 protein, compounds having an acidic functional group were screened by a partial structure search using the carboxyl group, sulfonyl group, and phosphate group structures. Compounds with a phosphate group structure were selected by using the phosphate group structure as a query and were further subjected to a partial structure search using the component of “substructure filter” in Pipeline Pilot. For the pharmacophore screening, the conformations of each compound were generated using Conformation Import in MOE with default settings. The query for the pharmacophore screening was prepared from 4OLA (PDB ID) by selecting the anion of the phosphate moiety with the hydrogen bond acceptor, the aromatic ring of adenine, and the acceptor of the first position of adenine. Pharmacophore screening was performed using Pharmacophore Search in MOE with default settings.

A partial charge was added by the AM1 method. Docking simulation was performed using the alpha sphere and excluded volume-based ligand-protein docking (ASE Dock) in MOE. Potential setups were set as follows: Amber 10, EHT force field; Solvation energy, Born equation; dielectric value, 2. The Protein-Ligand Interaction Fingerprint (PLIF) of all docking poses was extracted. Filtering was performed using the interaction of the phosphate moiety with Lys533, adenine with Tyr529, and the first position of adenine with Thr526.

### SPR analysis

SPR analysis was performed using Biacore T100 or T200 (GE Healthcare). The uridylic acid with biotin linker was chemically synthesized by GeneDesign (Osaka, Japan) and captured on the Series S Sensor Chip SA (GE Healthcare); 2.5 μg/mL of the AGO2 MID domain and each compound were co-added to the system using running buffer composed of HBS-EP+ (10 mmol/L HEPES [pH 7.4], 150 mmol/L NaCl, 3 mmol/L EDTA, 0.05% (v/v) Surfactant P20) (GE Healthcare), 2 mM dithiothreitol (DTT), and 5% dimethyl sulfoxide (DMSO). Data were analyzed using the Biacore Evaluation Software (GE Healthcare). Each resonance unit (RU) was normalized by that of buffer injection and the inhibition rate (%) was calculated using following formula: 100 x [1 - (RU/RU_witout compound_)].

### NMR analysis

All NMR experiments were carried out at 20°C on Bruker 600-MHz spectrometers (Bruker, Billerica, MA, USA) equipped with *z*-gradient triple resonance cryoprobes. An ^15^N-labeled AGO2 MID domain was dissolved in 25 mmol/L sodium phosphate, 150 mmol/L sodium chloride, and 2 mmol/L tris(2-carboxyethyl)phosphine (pH 6.70; 95% H_2_O/5% D_2_O). Backbone ^1^H_N_/^15^N chemical shift differences (Δ_H/N_) upon compound titrations were recorded on ^1^H-^15^N heteronuclear single-quantum coherence (HSQC) spectra. The spectra were processed using Topspin 3.1 (Bruker, Billerica, MA, USA) and analyzed using the program SPARKY (CA, USA) [22]. Δ_H/N_ was calculated in Hz at a ^1^H frequency of 600 MHz using the following formula: ([Δν_H_]^2^+[Δν_N_]^2^)^1/2^.

### RISC inhibition assay

HeLa cells (CCL-2) were directly purchased from American Type Culture Collection (ATCC) (Manassas, VA, USA). We created stock vials before use and finished the use of each vial within one month, though we have not tested fingerprinting in this study. Cells were maintained in Eagle’s minimum essential medium (ATCC) supplemented with 10% fetal bovine serum and 1% Zell shield (Minerva Biolabs, Berlin, Germany) at 37°C with 5% CO_2_. HeLa cells were collected by centrifugation and the cell pellet was lysed in a 2-pellet volume of lysis buffer (30 mmol/L HEPES-KOH [pH 7.4]), 100 mmol/L potassium acetate, 2 mmol/L magnesium acetate, 5 mmol/L DTT, and cOmplete™ EDTA-free protease inhibitor cocktail (Sigma-Aldrich) followed by Dounce homogenization [23,24]. Following centrifugation (17000 x*g*, 20 min at 4°C) to remove the nucleus, the supernatant was used as the HeLa lysate.

For immunoprecipitation, anti-human AGO2 (FUJIFILM Wako Pure Chemical, Osaka, Japan) and mouse IgG1κ isotype control antibody (Biolegend, San Diego, CA, USA) were captured, respectively, with Dynabeads protein G (Thermo Fisher Scientific, Waltham, MA, USA) using cell lysis buffer M (FUJIFILM Wako Pure Chemical).

The double-stranded RNA was chemically synthesized by GeneDesign (Osaka, Japan). The sequence of the sense strand was 5′-GGAUAGCAAGACCGACUAUCA-3′, while that of the antisense strand was 5′-AUAGUCGGUCUUGCUAUCCAU-3′. The 5’ terminus of the antisense strand was phosphorylated. The *in vitro* RISC assembly assay contained 5 μL of HeLa cell lysate, 3 μL of 40x reaction mix (containing ATP, ATP regeneration system, and RNase inhibitor) [23,24], 1 μL of 100 pM double-strand RNA, and 1 μL of a small molecule for 30 min at 25°C. The final reaction volume was 10 μL. DMSO was used for the baseline sample. After the reaction, 190 μL of 1% protease inhibitor cocktail/cell lysis buffer M and 5 μL of antibody-beads solution were added for 1.5 h at 4°C. The beads were washed four times using cell lysis buffer M and lysed with 20 μL of lysis buffer in the Cells to Ct kit (Thermo Fisher Scientific) for 10 min at room temperature. The reaction was stopped by adding 2 μL of stop solution in the Cells to Ct kit.

### Quantitative PCR

The primers and TaqMan probe were synthesized by Sigma-Aldrich (St. Louis, MO, USA) for quantitative PCR (qPCR) and Thermo Fisher Scientific, respectively. The extracted RNA and standard RNA were converted to cDNA using the TaqMan MicroRNA RT Kit (Thermo Fisher Scientific) with the following primer according to the manufacturer’s protocol: 5′-GTCGTATCCAGTGCAGGGTCCGAGGTATTCGCACTGGATACGACATGGAT-3′. The qPCR reaction was performed using TaqMan Gene Expression Master Mix (Thermo Fisher Scientific) using the following primers: forward: 5′-TAGTCGGTCTTGCTATC-3′ and reverse: 5′-GTGCAGGGTCCGAGGT-3′ and TaqMan probe (5′-CTGGATACGACATGGA-3′). Each threshold cycle (Ct value) was calculated using the QuantStudio™ 12K Flex Real-Time PCR System (Thermo Fisher Scientific).

### Cell viability analysis

HeLa cells were seeded on 96-well plates at 10,000 cells/well and each compound was added for 24 h at 37°C. The cell viability was measured using CellTiter-Glo® Luminescent Cell Viability Assay (Promega, Madison, WI, USA) according to the manufacturer’s protocol. Luminescence was measured as counts per second (CPS) for each well was read by ARVO-X3 (PerkinElmer, Waltham, MA, USA).

### Statistical analysis

Statistical analysis was performed with SAS software version 9.4. To compare differences between BCI-137 and each compound, one-way analysis of variance (1-way ANOVA) and Dunnett’s test were used. P values of < 0.05 were considered to indicate statistical significance.

## Results

### *In silico* SCR

**Fig 1:**
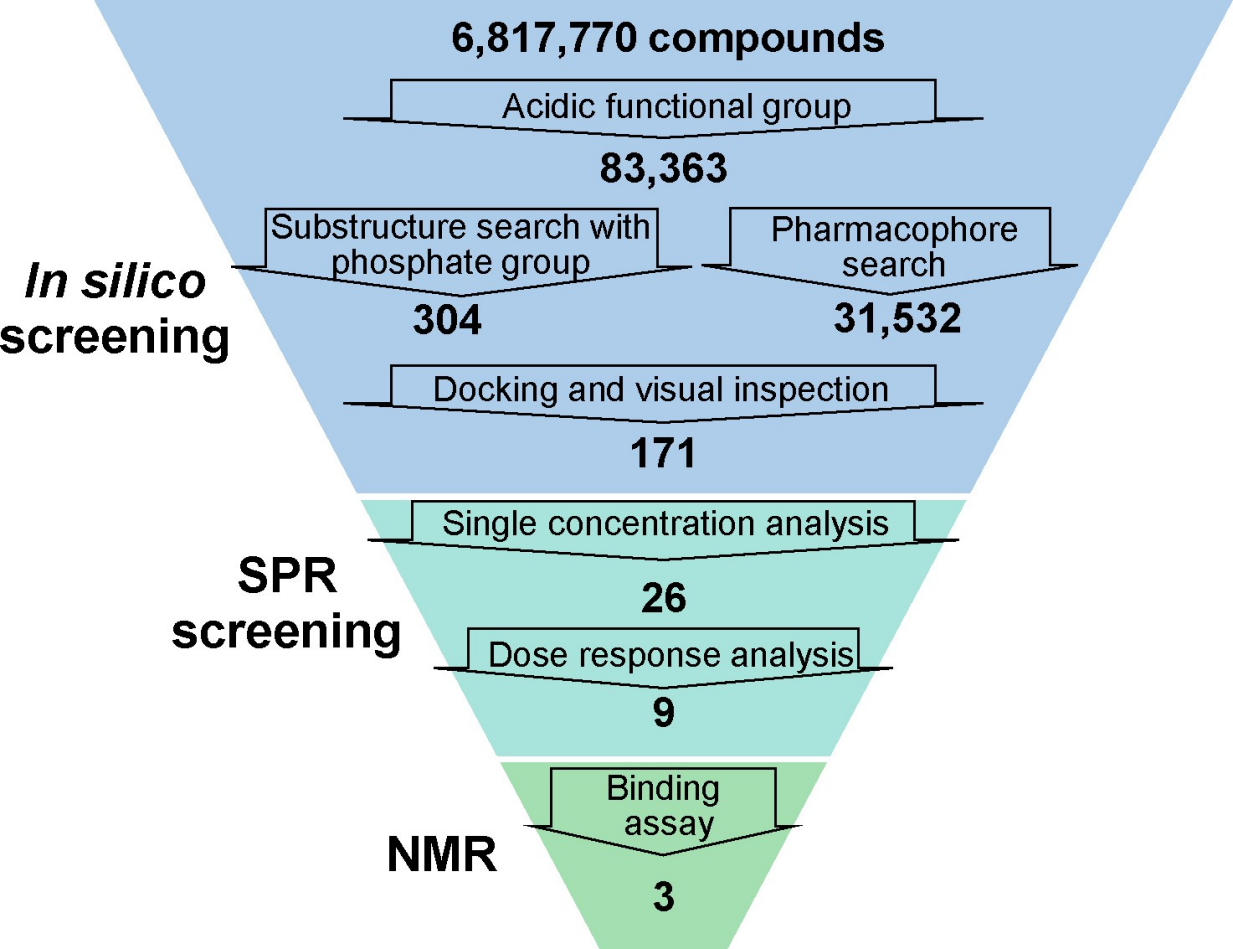
Workflow of screening. NMR, nuclear magnetic resonance; SPR, surface plasmon resonance.

Fig 1 summarizes the overall screening workflow. Step 1 involved *in silico* SCR. A total of 6,817,770 compounds from the chemical library were subjected to a partial structure search based on the carboxyl group, sulfonyl group, and phosphate group structures; of these, 83,363 compounds were selected. Since the probability of acquisition of “hits” might have been improved by selection based on multiple options, both the substructure search focusing on the phosphate group and structure-based screening were conducted. A partial structure search was performed using the phosphate group as a query, and 304 of the 83,363 compounds were screened. A structure-based method using the crystal structure of AGO2 was adopted for the pharmacophore screening, which identified 31,532 compounds. Thereafter, the selected compounds were subjected to the docking simulation, and in order to identify compounds that bind with a stronger affinity than AMP, two criteria were set: (1) the binding site should be the same as the 5’ terminus of RNA, and (2) more interactions should be observed than with AMP. Following the docking simulation, the compound poses were filtered by using the AMP interaction and visual inspection method, and 171 compounds were identified as “hits” using *in silico* SCR (S1 Table).

### Binding evaluation

**Fig 2:**
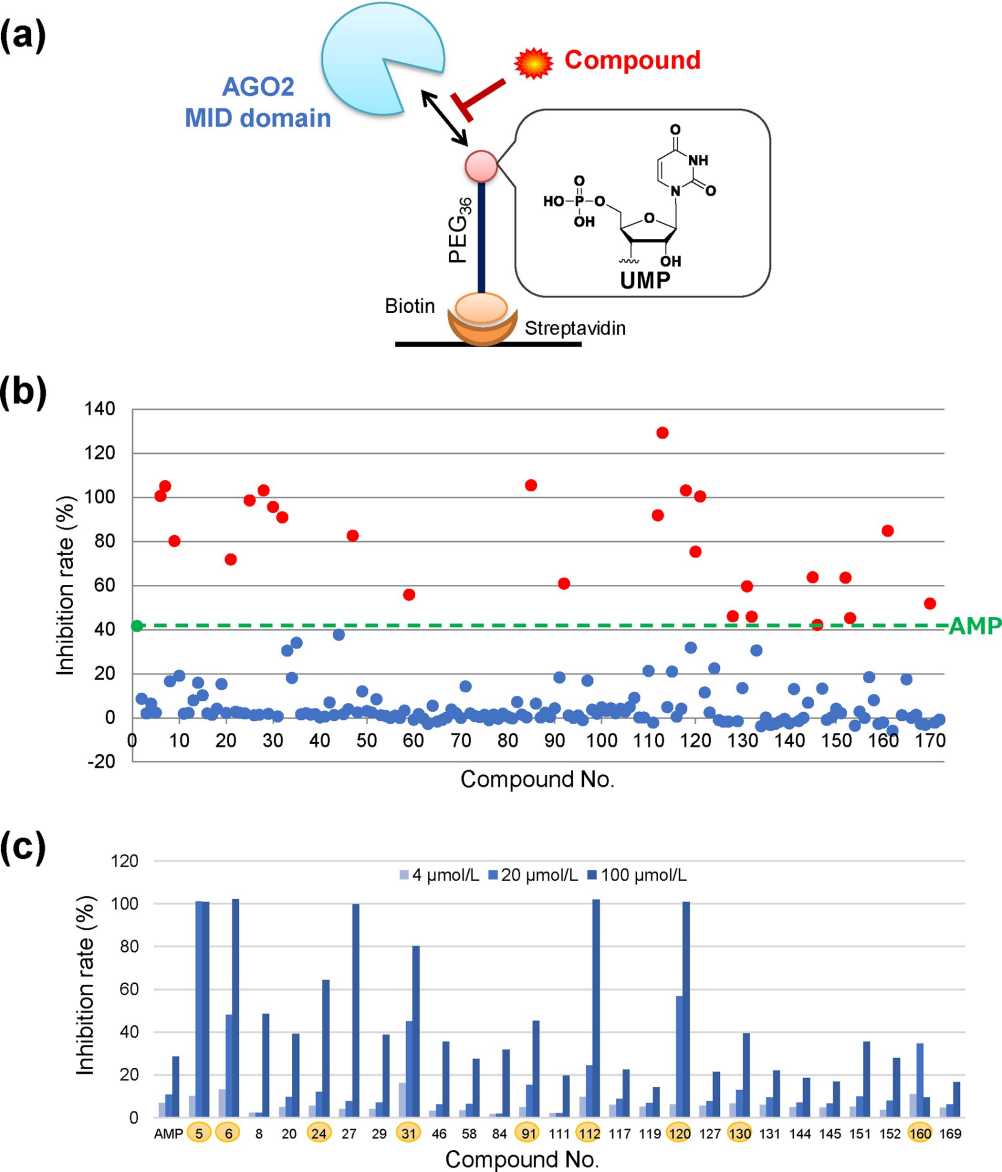
SPR screening focusing on the binding between the 5’ terminus of RNA and MID domain. (a) Experimental system of SPR screening. (b) Inhibition rate of 171 compounds obtained by *in silico* screening at 200 μmol/L. Green line indicates the criteria (inhibition rate of AMP). Red points indicate the “hit” candidates of the dose-response analysis. Blue points indicate unselected compounds. (c) Inhibition rate of 26 compounds at three concentrations. Highlighted compounds indicate the “hit” compounds from this screening. AGO, Argonaute; AMP, adenosine monophosphate; MID, middle; PEG, polyethylene glycol; SPR, surface plasmon resonance; UMP, uridine monophosphate.

In order to analyze the affinity of each screened compound to the AGO2 MID domain, we constructed an SPR inhibition system (Fig 2A). The UMP-harboring biotinylated polyethylene glycol (PEG) linker were immobilized on the streptavidin-immobilized sensor chip. The inhibitory activity was evaluated by co-adding the AGO2 MID domain with each compound.

In order to validate the evaluation system, we first analyzed the natural nucleotides. It has been reported that the affinity of UMP and AMP for the MID domain is stronger than that of CMP and GMP [16]. In order to analyze this base selectivity, GMP, CMP, AMP, and UMP were added as inhibitors to the system. In addition, the 5′-terminal phosphate has been shown to be critical for binding because the phosphate binds to Y529, K533, N545, and K566 of the AGO2 MID domain [19]. Furthermore, adenosine was added to confirm the importance of the 5'-terminal phosphate. The inhibition rate of each sample is shown in S1 Fig. AMP and UMP showed a stronger inhibition than CMP and GMP, demonstrating the ability of this system to distinguish the base selectivity. In addition, adenosine showed little inhibition (under 10% inhibiton at 500 μmol/L) because it has no terminal phosphate. These data indicated that the new evaluation system successfully analyzes the binding between the 5’ terminus of RNA and the AGO2 MID domain.

Next, 171 compounds screened by *in silico* SCR were analyzed at 200 μmol/L, which is near the half-maximal inhibitory concentration (IC50) of AMP. Compound 162 was excluded due to strong aggregation (Fig 2B); subsequently, 26 compounds showing stronger activity than AMP were selected as candidates (S2 Table). Thereafter, a dose response analysis was performed at lower concentrations (4 μmol/L, 20 μmol/L, and 100 μmol/L). These concentrations were set because AMP showed dose-dependent activity (S1 Fig). Eight compounds with stronger activity than AMP at 100 μmol/L and 20 μmol/L were selected. Compound 160 showed reduced activity at 100 μmol/L, but was selected due to the strong inhibition at 4 μmol/L and 20 μmol/L (Fig 2C).

**Fig 3:**
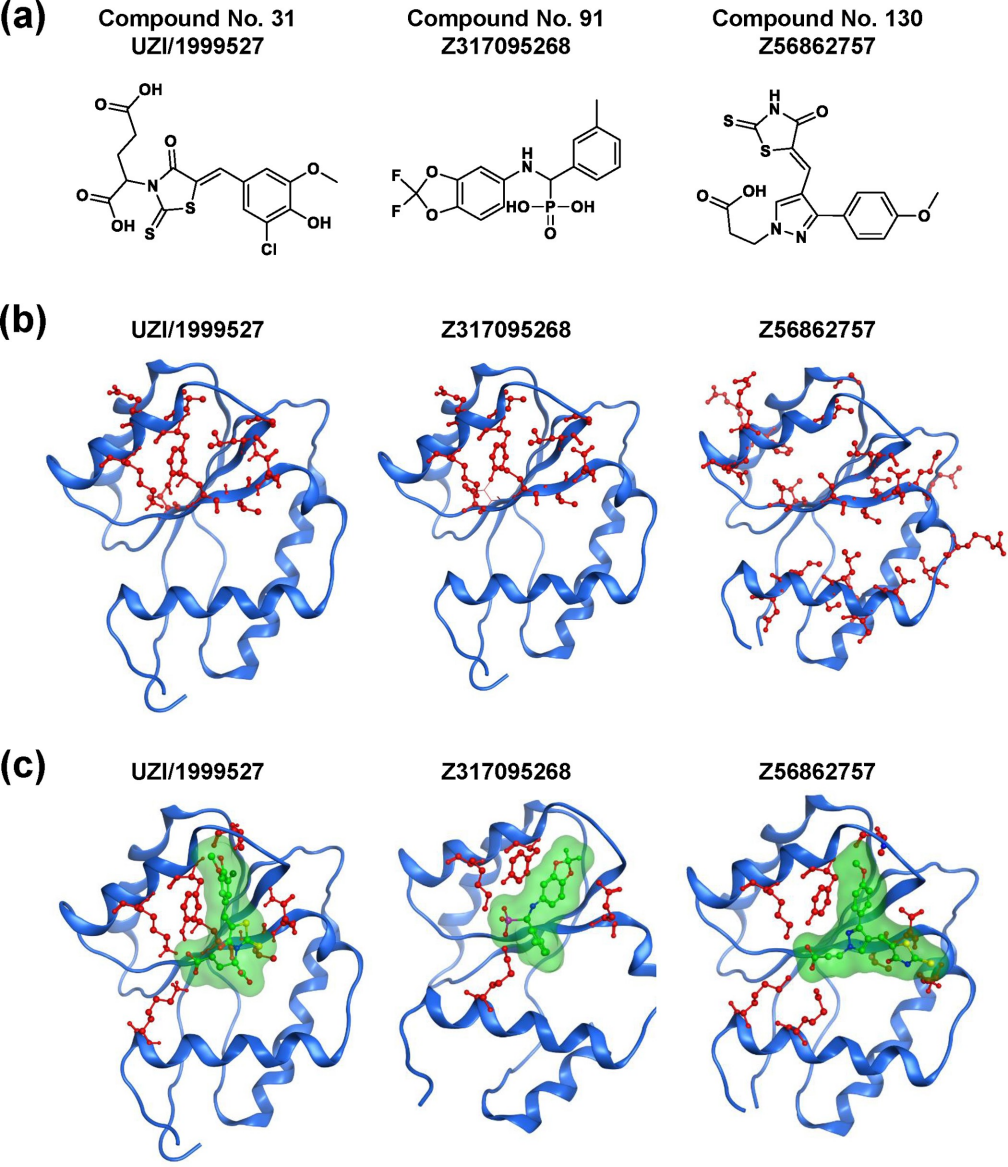
NMR binding evaluation. (a) Structure of hit compounds identified by NMR binding assay (E/Z isomer undetermined). (b) NMR signals showing CSP >30 Hz at a ratio = 5 are mapped on the crystal structure structure in red color with ball and stick representation (PDB ID: 3LUC). (c) Docking pose of each compound with the AGO2 MID domain. AGO, Argonaute; CSP, chemical shift perturbation; MID, middle; NMR, nuclear magnetic resonance; PDB, protein data bank.

Further analysis was carried out by NMR to evaluate whether the compounds bind to the nucleotide binding pocket in the AGO2 MID domain. NMR signal assignments from a chemical shift perturbation (CSP) experiment were used to predict a compound binding site, and three out of the nine compounds were confirmed (Fig 3A, S2–S4 Figs) to have perturbed amide chemical shifts of AGO2 MID domain backbone residues. Compound 160 also showed binding around the 5’ terminus of RNA. However, the range of CSP was limited, and binding near the 5′-terminal phosphate-binding site was not detected. Other compounds showed no binding to the AGO2 MID domain prior to compound precipitation. Fig 3B shows residues with a large chemical shift perturbation upon compound titration. Three of the hit compounds showed stronger inhibition than BCI-137 in the SPR analysis (S5 Fig).

**Fig 4:**
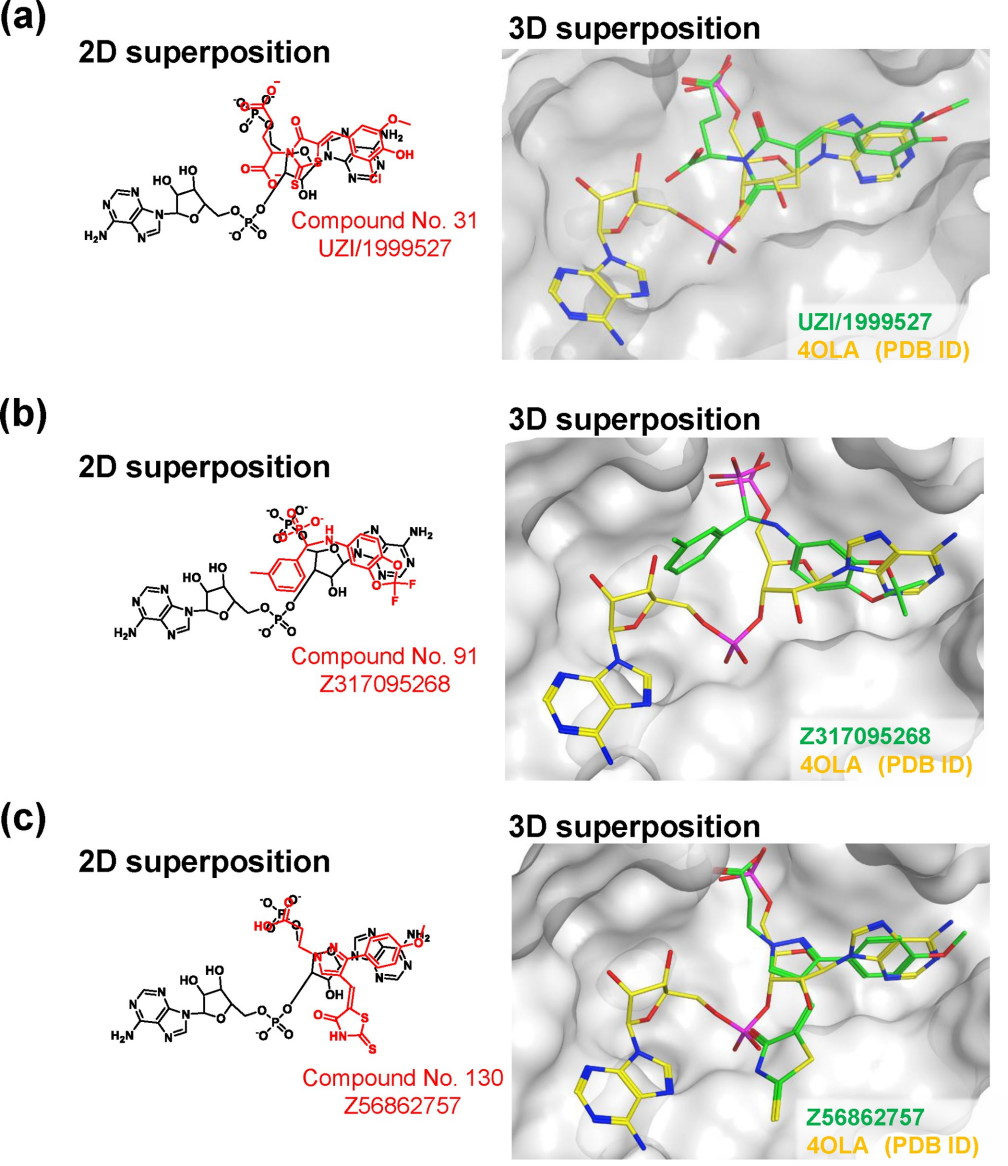
Docking mode of hit compounds compared with AMP structure. Superimposition of each hit compound and the 5' end of 4OLA (PDB ID). (a) UZI/1999527, (b) Z317095268, and (c) Z56862757. In 2D superposition, AMPs are shown in black; hit compounds are shown in red. In 3D superposition, AMPs are shown in yellow; hit compounds are shown in green. AMP, adenosine monophosphate.

Residues with a large chemical shift perturbation upon compound titration were compared with interacting residues in docking mode (Fig 3C). Although the docking pose of UZI/1999527 with the AGO2 MID domain was considered to have two candidates, we selected the one that matched with the NMR result. The binding mode of Z317095268 is consistent with the NMR results. Although NMR analysis suggests two Z56862757 binding sites, only one site could overlap with AMP. The overlapping structures of each compound with AMP are shown in Fig 4.

### RISC inhibition assay

**Fig 5:**
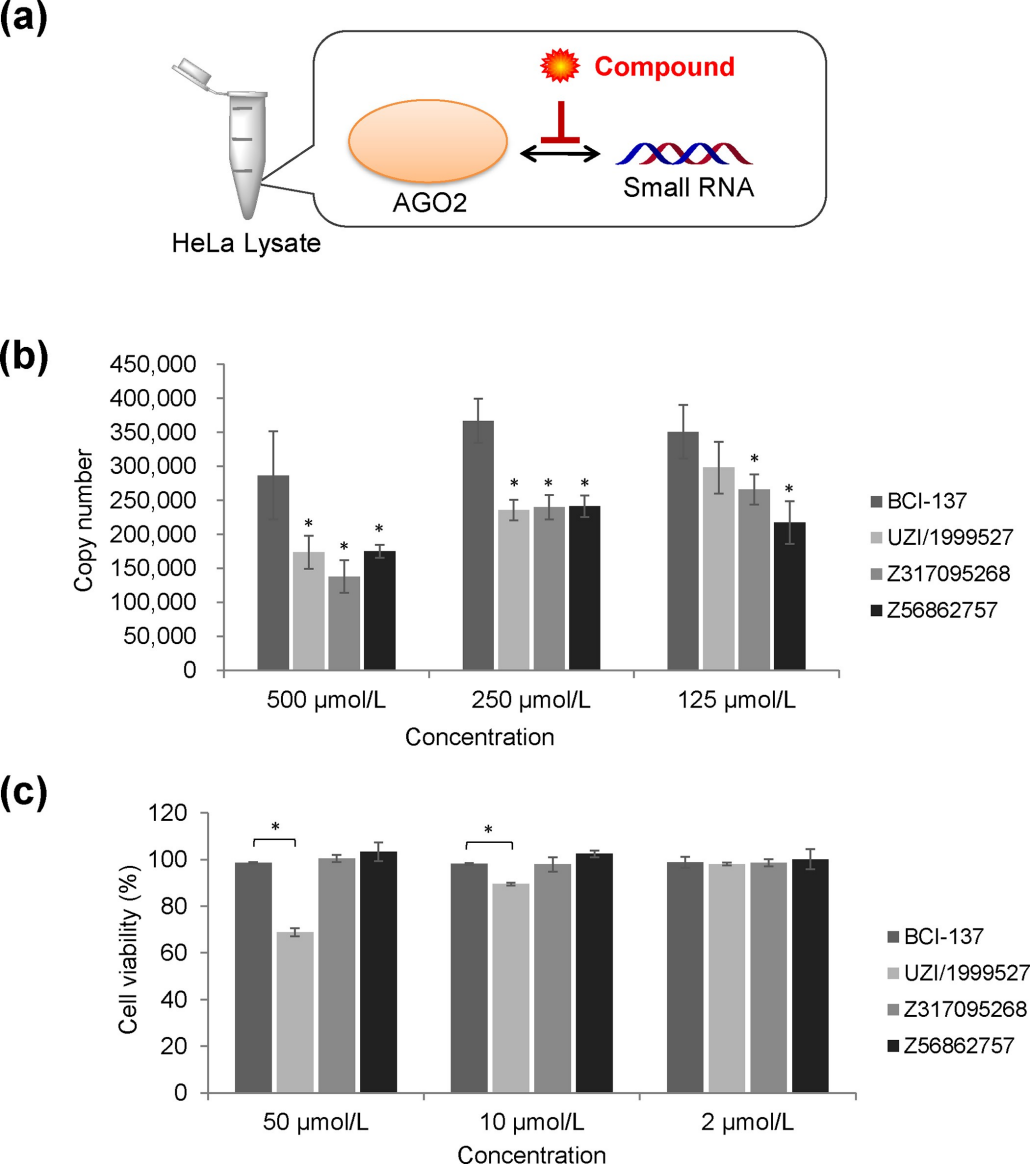
Biological activity of hit compounds. (a) Experimental system of RISC inhibition assay. (b) Number of AGO2-bound antisense strands analyzed by qPCR. The values represent the mean ± SD of triplicate experiments. (c) Cell viability of HeLa cells 24 h after the addition of each compound. The values represent the mean ± SD of triplicate experiments. AGO, Argonaute; DMSO, dimethyl sulfoxide; qPCR, quantitative polymerase chain reaction; RISC, RNA-induced silencing complex; SD, standard deviation; *, P < 0.05 versus BCI-137 group (one-way ANOVA followed by Dunnett’s test).

In order to evaluate the RISC inhibitory activity, each hit compound and double-stranded RNA was co-incubated in a HeLa lysate (Fig 5A). Luciferase-targeting siRNA was used as the model double-stranded RNA. After the reaction, AGO2 was immunoprecipitated and the antisense strand was quantified by qPCR. The specificity of immunoprecipitation was confirmed comparing with isotype control antibody. Fig 5B shows the number of AGO2-bound antisense strands. All three compounds successfully inhibited the binding between AGO2 and siRNA stronger than BCI-137. At the same time, we analyzed the amount of AGO2-bound miRNA to investigate the effect on endogenous miRNA. We chose miR-16 since it is one of the most stable-expressed housekeeping miRNAs. However, they did not show any inhibition to miR-16.

### Cytotoxicity of hit compounds

Each hit compound was added to HeLa cells to evaluate the influence on cell viability. The viability of HeLa cells 24 h after the addition of compound was measured (Fig 5C). While no inhibition was observed with Z317095268, Z56862757, and BCI-137, UZI/1999527 inhibited cell viability at 10 μmol/L and 50 μmol/L.

## Discussion

In this study, we identified new AGO2-binding compounds from a large chemical library by combining *in silico* SCR, SPR analysis, and NMR analysis. We focused on the strong binding between the 5’ terminus of RNA and the AGO2 MID domain, particularly the terminal phosphate. First, compounds with an acidic functional group were selected using a substructure search and structure-based screening. In SPR screening, we established a new inhibition evaluation system using the MID domain (one of the four domains of AGO2) and the UMP-harboring PEG linker. This *in vitro* system is simple, focuses only on the target site, and can be used for largescale screenings. NMR analysis revealed amino acid residues that contributed to the binding. Subsequently, three hit compounds that bound to the desired site with stronger affinity than BCI-137 were identified.

The RISC inhibitory activity was examined using HeLa lysate and siRNA. Inhibition of AGO2-bound antisense strand was observed, strongly suggesting that binding of hit compounds to the target site resulted in this RISC inhibitory activity. In addition, all three compounds showed equal or stronger inhibitory activity than BCI-137. This result is considered to correlate with the binding affinity. On the other hand, no significant inhibition of endogenous miR-16 was observed. It is reported that RISC assembly is not simple and divided into at least two steps: loading and maturation [25]. In loading step, double-stranded RNA binds to AGO with the help of chaperones. The resultant complex is called pre-RISC and yet unstable. After wedging and passenger ejection, the most stable state, mature RISC forms. Thus, removal of miRNA from mature RISC is considered to need higher energy than the inhibition of pre-RISC. In fact, an *in vitro* study showed that the antisense strand can be removed only by a highly complementary RNA [26]. Therefore, our results indicates that further affinity is required to remove endogenous miRNA from mature RISC.

We subsequently investigated the effect on cell viability. For the application such as improving retinoic acid-induced myeloid differentiation and virus suppression, toxicity should be avoided. Out of three hit compounds, UZI/1999527 inhibited the viability of HeLa cells. At this point, it is indistinguishable whether it inhibited cell growth or induced cell death because HeLa is cancer cells. It is expected that the cell viability analysis of normal cells or the evaluation of cell death clarify this toxicity. Moreover, we evaluated RISC inhibitory activity on luciferase-expressing HeLa cells. However, these compounds did not inhibit the luciferase-targeting siRNA in HeLa cells. Since all hit compounds had an acidic functional group, repulsion with cell membrane could have occurred. Thus, the development of a prodrug that masks the acidic functional group should be further explored.

In conclusion, we identified three compounds that strongly bind to AGO2 by combining an *in silico* docking study, SPR analysis, and NMR analysis. All hit compounds successfully inhibited the loading of siRNA in HeLa lysate. This screening strategy based on the crystal structure is useful to identify compounds for a nucleic acid-protein binding pocket. In order to enhance the affinity, fragment-based drug discovery (FBDD) approaches can be effective [27]. Since the docking mode of each compound was obtained, fragment growing or merging strategies will lead to more potent compounds.

## Supporting information

S1 Table List of 171 hit compounds screened by *in silico* SCR. SCR, combinatorial screening.
(PDF)

S2 Table List of 26 compounds screened by SPR. SPR, surface plasmon resonance.
(PDF)

S1 Fig SPR analysis of natural nucleoside and nucleotides. (a) Inhibition rate of natural nucleoside and nucleotides. The values represent the mean ± SD of triplicate experiments. (b) IC30 and IC50 values of each compound. Dose response curves of percent activity were fit using a four parameter logistic equation with the XLfit software program and each value were calculated. AMP, adenosine monophosphate; CMP, cytidine monophosphate; GMP, guanine monophosphate; IC, inhibitory concentration; N.D., not determined; SD, standard deviation; SPR, surface plasmon resonance; UMP, uridine monophosphate.
(TIF)

S2 Fig Overlay of ^1^H-^15^N HSQC spectra for ^15^N-AGO2 MID/UZI/1999527. Chemical shift perturbation (CSP) is recorded on the ^1^H-^15^N HSQC spectra of the AGO2 MID domain by titration of compound (top right structure). Each titration spectrum is overlaid at the molar ratio of [compound]/[AGO2 MID] = 0, 1, 5, and 15 in red, yellow, green, and cyan, respectively. AGO, Argonaute; HSCQ, heteronuclear single-quantum coherence; MID, middle.
(TIF)

S3 Fig Overlay of ^1^H-^15^N HSQC spectra for ^15^N-AGO2 MID/Z317095268. Chemical shift perturbation (CSP) is recorded on the ^1^H-^15^N HSQC spectra of the AGO2 MID domain by titration of compound (top right structure). Each titration spectrum is overlaid at the molar ratio of [compound]/[AGO2 MID] = 0, 1, 5, and 15 in red, yellow, green, and cyan, respectively. AGO, Argonaute; HSCQ, heteronuclear single-quantum coherence; MID, middle.
(TIF)

S4 Fig Overlay of ^1^H-^15^N HSQC spectra for ^15^N-AGO2 MID/Z56862757. Chemical shift perturbation (CSP) is recorded on the ^1^H-^15^N HSQC spectra of the AGO2 MID domain by titration of compound (top right structure). Each titration spectrum is overlaid at the molar ratio of [compound]/[AGO2 MID] = 0, 1, 5, and 15 in red, yellow, green, and cyan, respectively. AGO, Argonaute; HSCQ, heteronuclear single-quantum coherence; MID, middle.
(TIF)

S5 Fig SPR analysis of hit compounds and BCI-137. (a) Inhibition rate of hit compounds and BCI-137. The values represent the mean ± SD of triplicate experiments. (b) IC30 values of each compound. Dose response curves of percent activity were fit using a four parameter logistic equation with the XLfit software program and IC30 value were calculated. The values represent the mean ± SD of triplicate experiments. IC, inhibitory concentration; N.D., not determined; SD, standard deviation; SPR, surface plasmon resonance.
(TIF)
