# Supplementary material for: Structure-guided screening strategy combining surface plasmon resonance with nuclear magnetic resonance for identification of small-molecule Argonaute 2 inhibitors
Source: PLoS One. 2020 Jul 31;15(7):e0236710. doi: 10.1371/journal.pone.0236710 (PMC7394379; doi:10.1371/journal.pone.0236710)
Supplement: S1 Table — SCR, combinatorial screening. (PDF) [file pone.0236710.s001.pdf]

No	Supplier	ID No.
1	App Tec	TD155034A_Y03
2	App Tec	WX120019_Y02A594
3	ASINEX	BAS 00513756
4	Vitas-M	STK047849
5	Vitas-M	STK152682
6	Vitas-M	STK153311
7	Vitas-M	STK156197
8	Vitas-M	STK186421
9	Vitas-M	STK223653
10	Vitas-M	STK247409
11	Vitas-M	STK267662
12	Vitas-M	STK295737
13	Vitas-M	STK295999
14	Vitas-M	STK296036
15	Vitas-M	STK312376
16	Vitas-M	STK312851
17	Vitas-M	STK328658
18	Vitas-M	STK436155
19	Vitas-M	STK465041
20	Vitas-M	STK465791
21	Vitas-M	STK466370
22	Vitas-M	STK466824
23	Vitas-M	STK466933
24	Vitas-M	STK669839
25	Vitas-M	STK678286
26	Vitas-M	STK691833
27	Vitas-M	STK810229
28	Vitas-M	STK976063
29	Zelinsky Institute	UZI/1416207
30	Zelinsky Institute	UZI/1634720
31	Zelinsky Institute	UZI/1999527
32	Zelinsky Institute	UZI/2536166
33	Zelinsky Institute	UZI/2603127
34	Zelinsky Institute	UZI/2645135
35	Zelinsky Institute	UZI/8154952
36	Enamine	EN300-00298
37	Enamine	EN300-00369
38	Enamine	PB115663836
39	Enamine	PB224222440
40	Enamine	PB224342264
41	Enamine	PB224354460
42	Enamine	PB224360154
43	Enamine	PB317356606
44	Enamine	PB45621479
45	Enamine	PB45814707
46	Enamine	PB46081736
47	Enamine	PB608651940
48	Enamine	PB85994517
49	Enamine	PB997840664
50	Enamine	Z1014322250
51	Enamine	Z1014322636
52	Enamine	Z1014323040
53	Enamine	Z107274704
54	Enamine	Z1136440592
55	Enamine	Z118438488
56	Enamine	Z1222429580
57	Enamine	Z1278811187
58	Enamine	Z1310787866
59	Enamine	Z1444440142
60	Enamine	Z1444642239
61	Enamine	Z1444832847
62	Enamine	Z1444967382
63	Enamine	Z1444972868
64	Enamine	Z1445236793
65	Enamine	Z1445442377
66	Enamine	Z1445568915
67	Enamine	Z1521553982
68	Enamine	Z1524550234
69	Enamine	Z1603232788
70	Enamine	Z1603469325
71	Enamine	Z1603611175
72	Enamine	Z1606913939
73	Enamine	Z1609539874
74	Enamine	Z1609605009
75	Enamine	Z17835123
76	Enamine	Z18519580
77	Enamine	Z19629328
78	Enamine	Z19629972
79	Enamine	Z19630022
80	Enamine	Z19690974
81	Enamine	Z223122150
82	Enamine	Z223122808
83	Enamine	Z223342720
84	Enamine	Z224280092
85	Enamine	Z227731180
86	Enamine	Z229639936
87	Enamine	Z242710880
88	Enamine	Z24799109
89	Enamine	Z295808060
90	Enamine	Z317088428
91	Enamine	Z317095268
92	Enamine	Z317437816
93	Enamine	Z324260754
94	Enamine	Z324266500
95	Enamine	Z353969056
96	Enamine	Z353983390
97	Enamine	Z365098478
98	Enamine	Z365098598
99	Enamine	Z365098682
100	Enamine	Z365098694
101	Enamine	Z365098910
102	Enamine	Z365100352
103	Enamine	Z365100460
104	Enamine	Z365101582
105	Enamine	Z365103426
106	Enamine	Z394668322
107	Enamine	Z398833274
108	Enamine	Z44303812
109	Enamine	Z44350081
110	Enamine	Z44840929
111	Enamine	Z44864370
112	Enamine	Z449724762
113	Enamine	Z45656071
114	Enamine	Z45679031
115	Enamine	Z45689159
116	Enamine	Z45735699
117	Enamine	Z45979706
118	Enamine	Z46008622
119	Enamine	Z46010227
120	Enamine	Z46149023
121	Enamine	Z49593944
122	Enamine	Z49723743
123	Enamine	Z49913090
124	Enamine	Z53037929
125	Enamine	Z53038375
126	Enamine	Z53113208
127	Enamine	Z56813902
128	Enamine	Z56816756
129	Enamine	Z56850507
130	Enamine	Z56862757
131	Enamine	Z56911979
132	Enamine	Z57038485
133	Enamine	Z71177768
134	Enamine	Z812953938
135	Enamine	Z812953974
136	Enamine	Z812953978
137	Enamine	Z85882597
138	Enamine	Z85888269
139	Enamine	Z85899722
140	Enamine	Z85916495
141	Enamine	Z915551068
142	Enamine	Z929457378
143	Enamine	Z929461746
144	Enamine	Z940507090
145	Enamine	Z940508482
146	Enamine	Z940509644
147	Enamine	Z978943702
148	Enamine	Z997748538
149	Enamine	Z997748570
150	Enamine	Z997755878
151	Enamine	Z997825328
152	Labotest	LT00452182
153	Labotest	LT00724247
154	Labotest	LT01322127
155	Life Chemicals	F5038-0199
156	MAYBRIDGE	HTS09249
157	Scientific Exchange	M-582672
158	Scientific Exchange	X-051539
159	BIONET	RS-0040
160	Combi-Blocks	SS-9182
161	Pharmeks	P2000S-19570
162	Pharmeks	P2001S-023258
163	Enamine	Z1041114094
164	Enamine	Z131082802
165	Enamine	Z324257098
166	Enamine	Z324258758
167	Enamine	Z324259756
168	Enamine	Z359420382
169	Enamine	Z46025276
170	MAYBRIDGE	RJC02525
171	Pharmeks	P2001S-431079
