# Supplementary material for: Structure-guided screening strategy combining surface plasmon resonance with nuclear magnetic resonance for identification of small-molecule Argonaute 2 inhibitors
Source: PLoS One. 2020 Jul 31;15(7):e0236710. doi: 10.1371/journal.pone.0236710 (PMC7394379; doi:10.1371/journal.pone.0236710)
Supplement: S2 Table — SPR, surface plasmon resonance. (PDF) [file pone.0236710.s002.pdf]

No.	Supplier	ID No.	Structure	No.	Supplier	ID No.	Structure
5	Vitas-M	STK152682	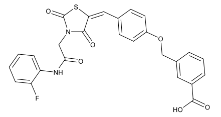	112	Enamine	Z449724762	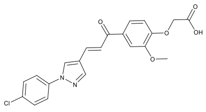
6	Vitas-M	STK153311	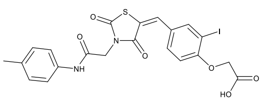	117	Enamine	Z45979706	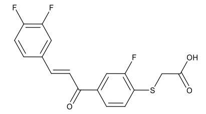
8	Vitas-M	STK186421	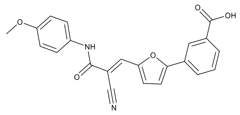	119	Enamine	Z46010227	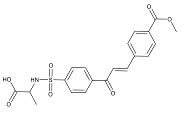
20	Vitas-M	STK465791	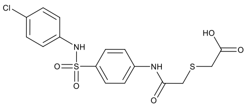	120	Enamine	Z46149023	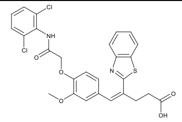
24	Vitas-M	STK669839	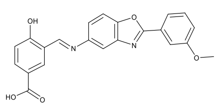	127	Enamine	Z56813902	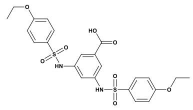
27	Vitas-M	STK810229	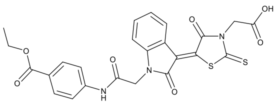	130	Enamine	Z56862757	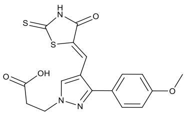
29	Zelinsky Institute	UZI/1416207	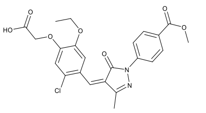	131	Enamine	Z56911979	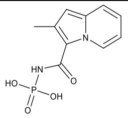
31	Zelinsky Institute	UZI/1999527	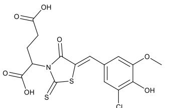	144	Enamine	Z940507090	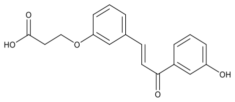
46	Enamine	PB46081736	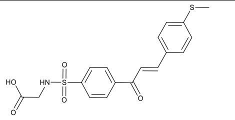	145	Enamine	Z940508482	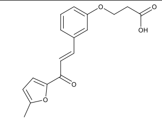
58	Enamine	Z1310787866	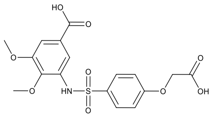	151	Enamine	Z997825328	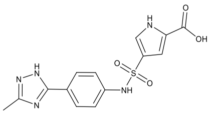
84	Enamine	Z224280092	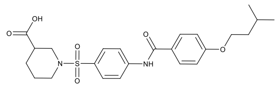	152	Labotest	LT00452182	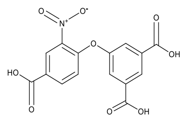
91	Enamine	Z317095268	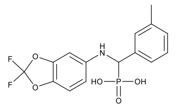	160	Combi-Blocks	SS-9182	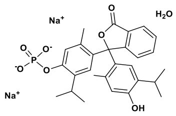
111	Enamine	Z44864370	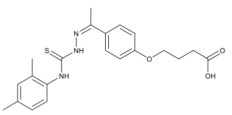	169	Enamine	Z46025276	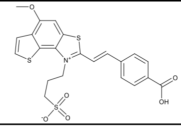
