# Supplementary figures and images for: Structure-guided screening strategy combining surface plasmon resonance with nuclear magnetic resonance for identification of small-molecule Argonaute 2 inhibitors
Source: PLoS One. 2020 Jul 31;15(7):e0236710. doi: 10.1371/journal.pone.0236710 (PMC7394379; doi:10.1371/journal.pone.0236710)

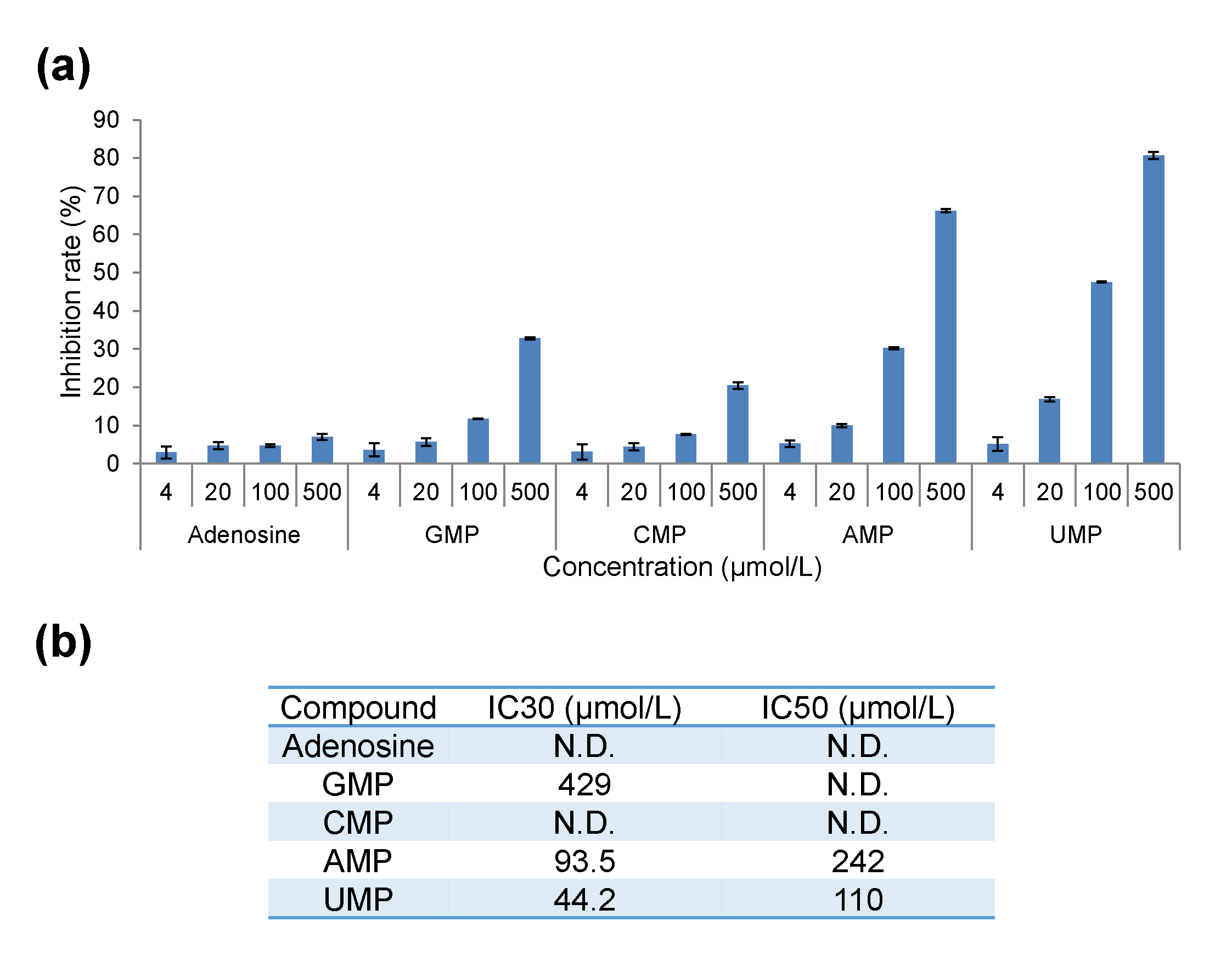

Supplement: S1 Fig — (a) Inhibition rate of natural nucleoside and nucleotides. The values represent the mean ± SD of triplicate experiments. (b) IC30 and IC50 values of each compound. Dose response curves of percent activity were fit using a four parameter logistic equation with the XLfit software program and each value were calculated. AMP, adenosine monophosphate; CMP, cytidine monophosphate; GMP, guanine monophosphate; IC, inhibitory concentration; N.D., not determined; SD, standard deviation; SPR, surface plasmon resonance; UMP, uridine monophosphate. (TIF) [file pone.0236710.s003.tif]

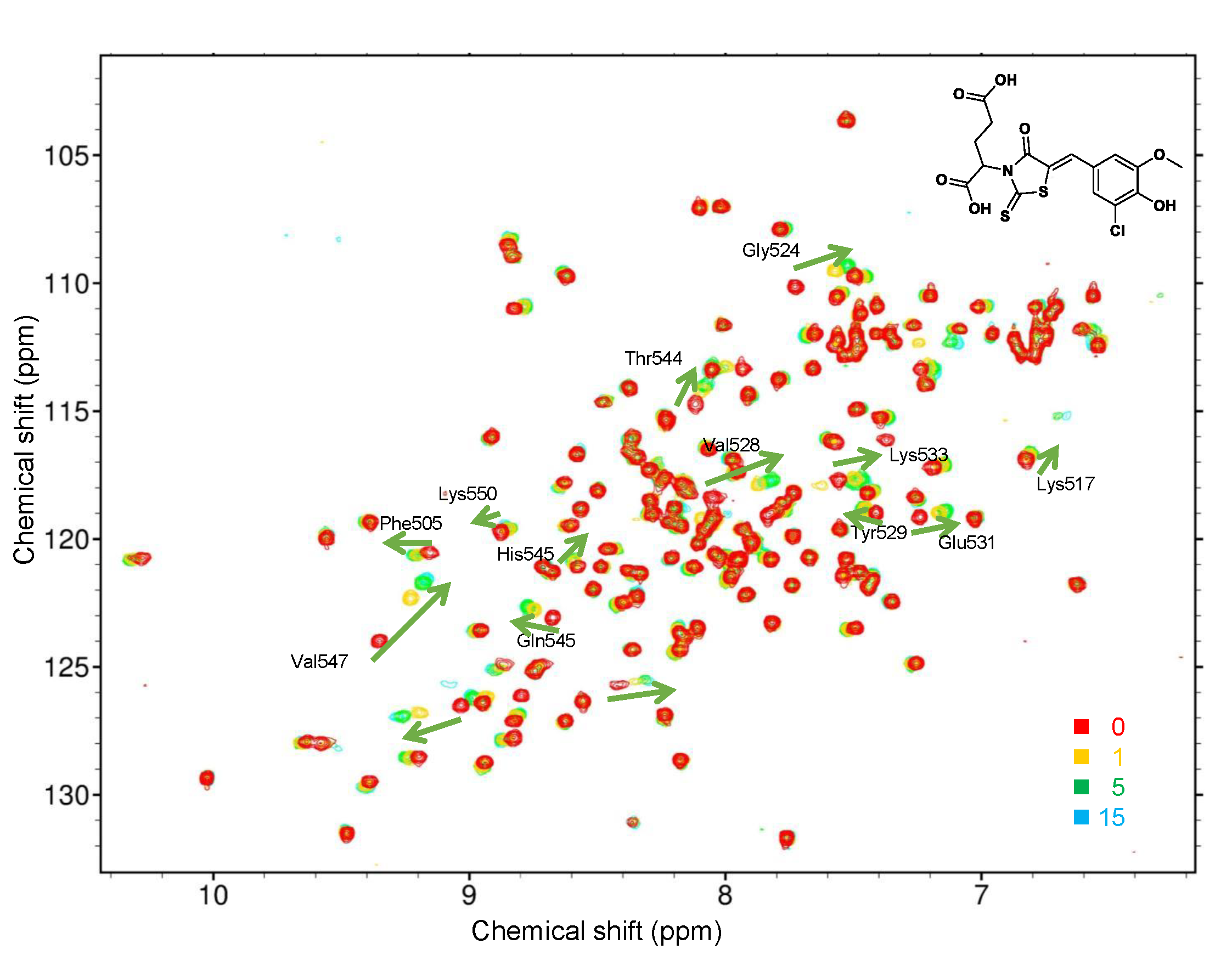

Supplement: S2 Fig — Chemical shift perturbation (CSP) is recorded on the 1H-15N HSQC spectra of the AGO2 MID domain by titration of compound (top right structure). Each titration spectrum is overlaid at the molar ratio of [compound]/[AGO2 MID] = 0, 1, 5, and 15 in red, yellow, green, and cyan, respectively. AGO, Argonaute; HSCQ, heteronuclear single-quantum coherence; MID, middle. (TIF) [file pone.0236710.s004.tif]

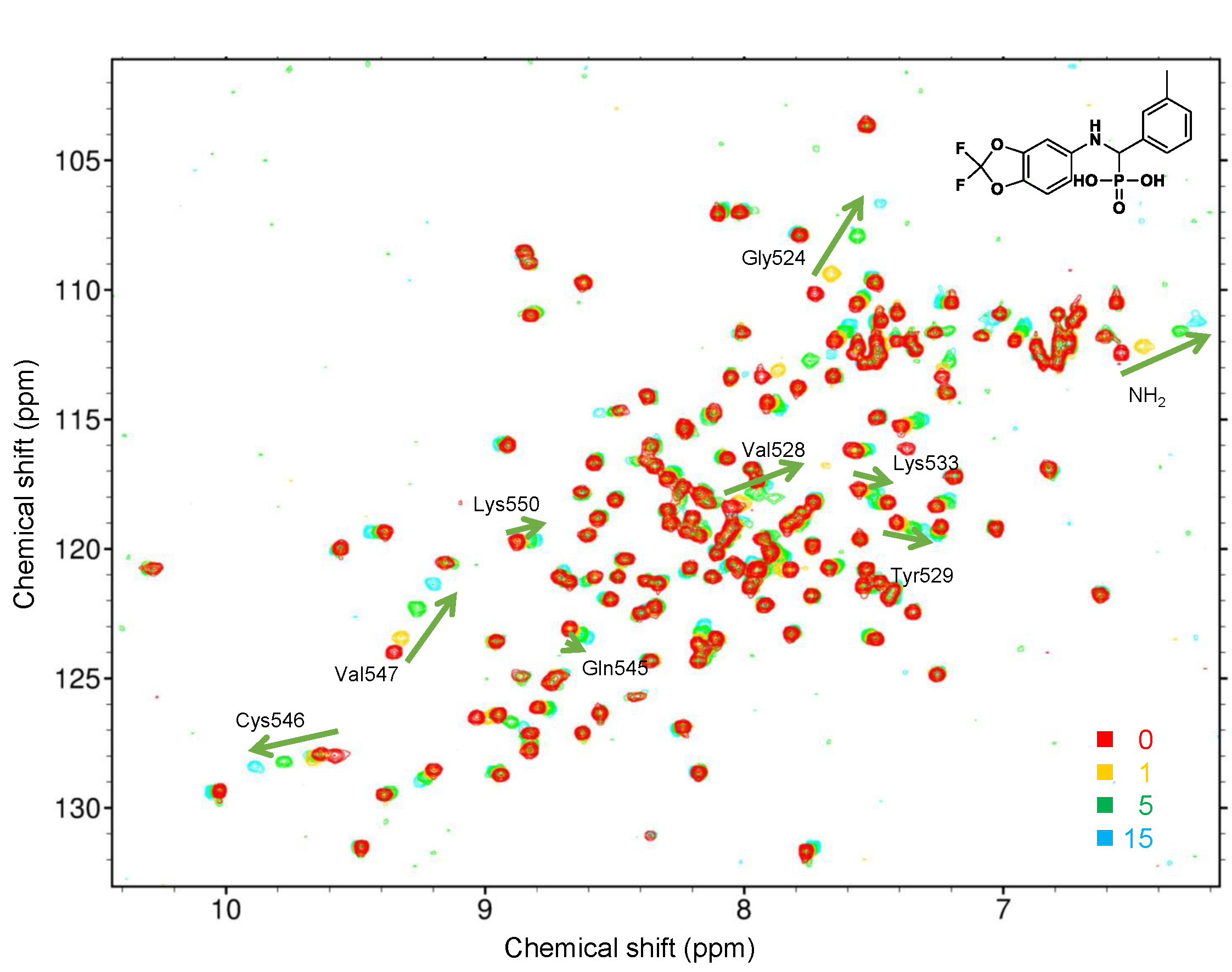

Supplement: S3 Fig — Chemical shift perturbation (CSP) is recorded on the 1H-15N HSQC spectra of the AGO2 MID domain by titration of compound (top right structure). Each titration spectrum is overlaid at the molar ratio of [compound]/[AGO2 MID] = 0, 1, 5, and 15 in red, yellow, green, and cyan, respectively. AGO, Argonaute; HSCQ, heteronuclear single-quantum coherence; MID, middle. (TIF) [file pone.0236710.s005.tif]

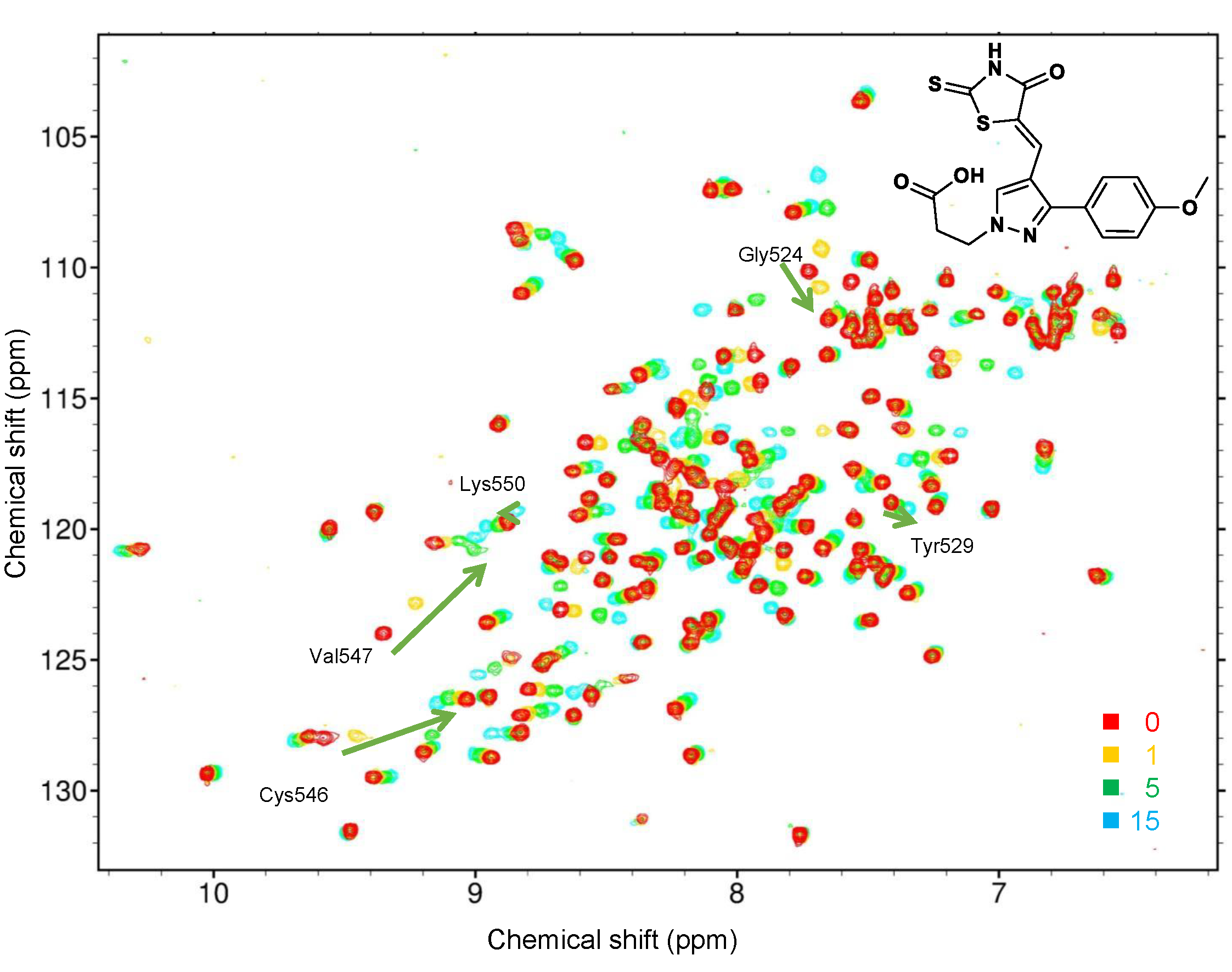

Supplement: S4 Fig — Chemical shift perturbation (CSP) is recorded on the 1H-15N HSQC spectra of the AGO2 MID domain by titration of compound (top right structure). Each titration spectrum is overlaid at the molar ratio of [compound]/[AGO2 MID] = 0, 1, 5, and 15 in red, yellow, green, and cyan, respectively. AGO, Argonaute; HSCQ, heteronuclear single-quantum coherence; MID, middle. (TIF) [file pone.0236710.s006.tif]

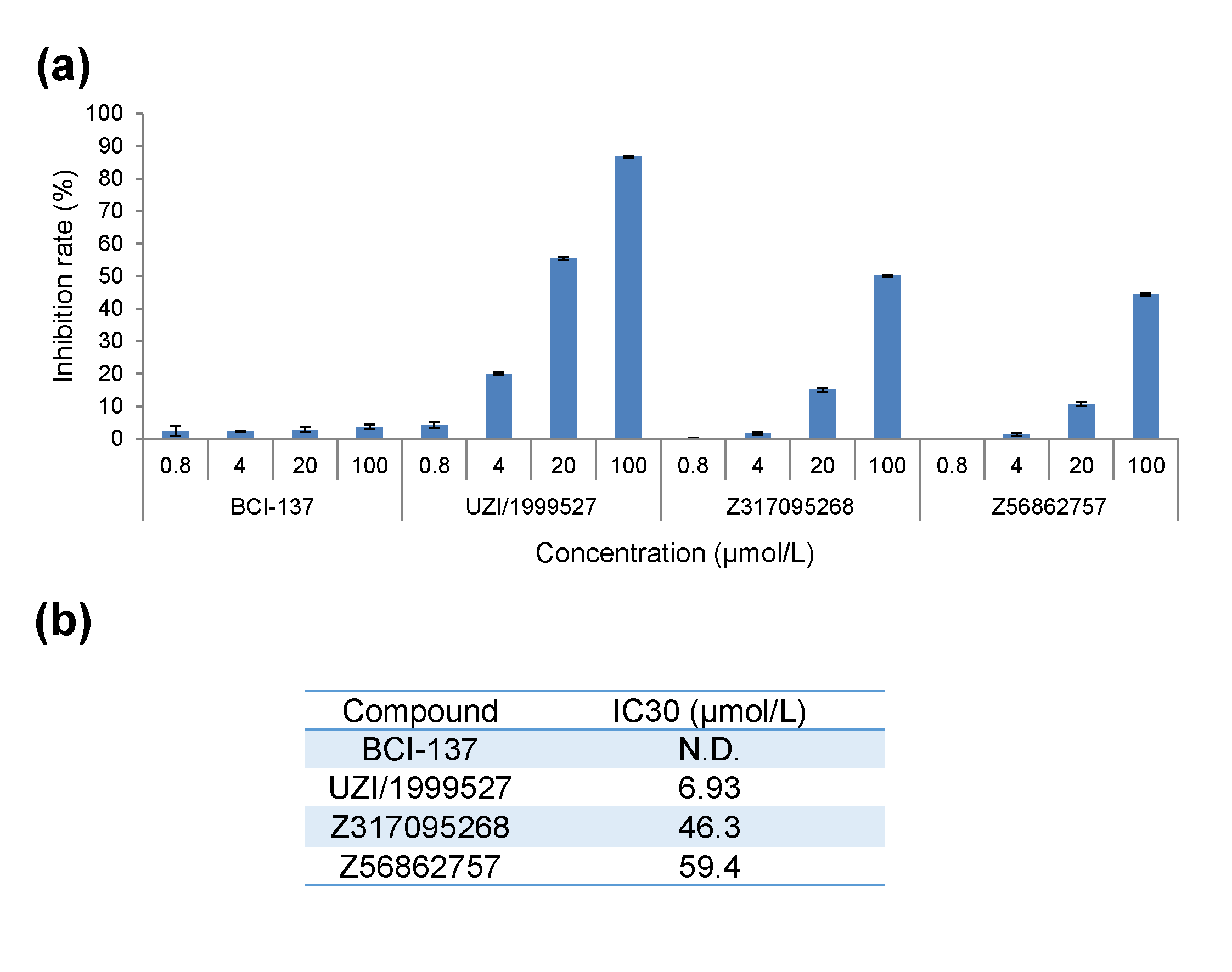

Supplement: S5 Fig — (a) Inhibition rate of hit compounds and BCI-137. The values represent the mean ± SD of triplicate experiments. (b) IC30 values of each compound. Dose response curves of percent activity were fit using a four parameter logistic equation with the XLfit software program and IC30 value were calculated. The values represent the mean ± SD of triplicate experiments. IC, inhibitory concentration; N.D., not determined; SD, standard deviation; SPR, surface plasmon resonance. (TIF) [file pone.0236710.s007.tif]
